# Severe COVID-19 classified by simple covid risk index is associated with higher levels of advanced oxidation protein products and 8-hydroxy 2 deoxyguanosine

**DOI:** 10.1017/S0950268823001280

**Published:** 2023-08-09

**Authors:** Joanna Satała, Agnieszka Woźniak, Mateusz Fabiś, Paulina Gorzelak-Pabiś, Agnieszka Pawlos, Jarosław Fabiś, Marlena Broncel, Ewelina Woźniak

**Affiliations:** 1 Department of Internal Diseases and Clinical Pharmacology, The Laboratory of Tissue Immunopharmacology, Medical University of Lodz, Lodz, Poland; 2 Department of Arthroscopy, Minimally Invasive Surgery and Sports Traumatology, Medical University of Lodz, Lodz, Poland

**Keywords:** AOPP, COVID-19, oxidative stress, SCRI, 8-OHDG

## Abstract

SARS-CoV-2 has become one of the most important and challenging medical research topics in recent years. The presence of endothelial dysfunction, immune thrombosis, and oxidative stress contributes to complications and requires more extended hospitalisation of patients. In this article, we focused on analysing the impact of oxidative stress on the severity of COVID-19 infection. The study group consisted of 72 patients with laboratory-confirmed SARS-CoV enrolled. The patients were divided into moderate and severe diseases according to the SCRI (Simple Covid Risk Index, including lymphocyte/D-dimer ratio). Using the ELISA kit, we determined the level of AOPP and 8-OHdG. Patients with severe COVID-19 had higher levels of both AOPP (*P* < 0.05) and 8-OHdG (*P* < 0.05) compared to patients with moderate disease. Albumin levels were significantly lower (*P* < 0.001), although fibrinogen (*P* < 0.01), D-dimer (*P* < 0.001), and TF (*P* < 0.05) levels were higher in severe patients than in moderate course. AOPP/Alb was also higher among severe patients (*P* < 0.05). Our data suggest a potential role for AOPP and 8-OHdG in predicting the outcome of SARS-CoV-2 patients. Elevated AOPP levels were associated with increased Dimer-D, TF, and vWF activity levels.

## Graphical abstract

**Figure:**
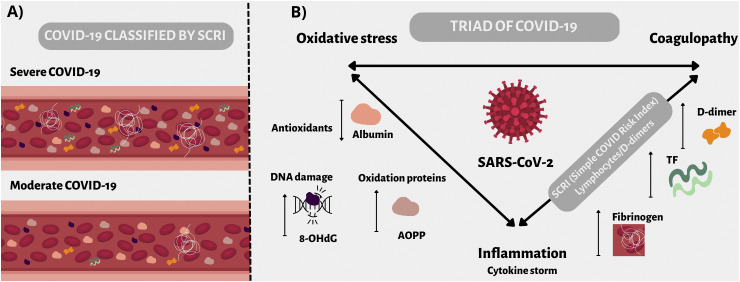

## Introduction

COVID-19 infection induces both immunological and thrombotic dysregulation. Simple Covid Risk Index (SCRI, lymphocytes/d-dimers count ratio), described based on our previous analysis, determines the disease correlated to the severity and can be calculated following hospital admission. The high sensitivity and specificity of SCRI suggest that it may be helpful in everyday clinical practice to predict whether COVID-19 patients would develop moderate or severe disease [1]. Recent studies showed that the pro-inflammatory cytokine storm in the severe course of COVID-19 could lead to CARDS (COVID-19-associated acute respiratory distress syndrome), damaging clinical effects, or even mortality among patients [2, 3]. Cytokine storm, inflammation, damage, and impairment of the infected endothelial cells are all components of COVID-19 but also connect with the development of oxidative stress [4]. Inflammation and oxidative stress have become two critical elements in COVID-19, leading to endothelial dysfunction, and exacerbating the disease [5]. Oxidative stress can be defined as an imbalance between the production of reactive oxygen species (ROS) and its removal by endogenous mechanisms (system of antioxidants). Biomarkers that can reflect the severity of oxidative stress in COVID-19 are advanced oxidation protein products (AOPP) and 8-hydroxy 2 deoxyguanosine (8OHdG) [6, 7].

AOPP are tyrosine-containing molecules mainly modified by oxidations of albumin, fibrinogen, and lipoproteins. Their level can reflect inflammation intensity, including activation of monocytes and neutrophils [8]. Accumulation of excessive AOPP during lung injury can initiate decreased lung fluid clearance, contributing to the disease progression [9].

The second marker, 8-OHdG, is a widely abundant oxidative lesion of DNA. The concentration of the 8-OHdG is associated with the actual level of fragmentation and modification of DNA caused by ROS. During the DNA replication, the oxo- group and the hydrogen atom are introduced into the imidazole ring of dG, leading to premutagenic dV: dG to dA:dT transversion [10]. Therefore, an increase in 8-OHdG may correlate with the extent of damage in various lung pathologies [11].

We hypothesised that oxidative stress expressed as the concentration of its biomarkers could be related to the exacerbation and severity of COVID-19. In our previous work, we described an innovative prognostic indicator for COVID-19 – SCRI, which is innovative in that it considers the activation of the immune system (lymphocytes) and thromboembolic complications (D-dimer). We compare both groups and find that AOPP and 8-OHdG are higher in the severe group, based on the SCRI, suggesting that oxidative stress also contributes to the severity of the course along with the immune and thromboembolic systems. We also aimed to find correlations between oxidative stress factors and, inflammation and coagulation parameters.

## Methods

### Study population

This study included 72 adult patients who were diagnosed with COVID-19. Infection with SARS-CoV-2 was confirmed by a reverse polymerase chain reaction (RT-PCR) or antigen test in a nasopharyngeal swab. COVID-19 patients were hospitalised from April to June 2021 at the Department of Internal Diseases and Clinical Pharmacology at Bieganski Hospital. Routine blood samples were collected at admission from each patient. Clinical severity was assessed at admission, with the SCRI being used to divide the patients into two groups according to the course of COVID-19 [3]. The SCRI cut-off equals 1.19. Therefore, patients with a score of 1.19 or higher were categorised as moderate COVID-19 (*n* = 47) and those below 1.19 as severe (*n* = 25). The research was approved by the Bioethics Committee of the Medical University of Lodz No RNN/122/21/KE dated 13.04.2021.

### Materials

Blood samples were collected on the day of admission. All samples were processed within 2 hours of collection using a closed system. In order to obtain plasma, blood was collected in coagulation tubes with 3.8% sodium citrate and then centrifuged at 1500 g for 10 min. Plasma for tissue factor (TF) assay was isolated by centrifugation of the blood samples at 1000 g for 15 min., according to the producer’s protocol. The serum for most assays was obtained after centrifugation at 4000 g for 5 min, in serum separation tubes.

### Elisa

The concentration of AOPP was determined in plasma by the ELISA kit (Cloud-Clone Corp., USA). The minimum detectable level of AOPP was 50.4 pg/mL. Samples required about 50.000-fold dilution, which was achieved by a series of dilutions.

The quantitative measurement of the oxidative DNA adduct 8-OhdG in serum was achieved by enzyme-linked immunosorbent assay (JaICA., Japan). The samples required three-fold dilution. The minimum detectable concentration of 8-OHdG was 0.125 ng/mL.

### Blood tests

Neutrophils were counted in EDTA-anticoagulated blood on the DxH 900 Hematology Analyzer (DxH900, DxH800 Beckman Coulter, Japan). The concentration of Zinc (Zn) in serum was measured using the reference atomic absorption (AA) method (PinAAcle 900, PerkinElmer). Vitamin D was detected by electrochemiluminescence immunoassay (ECLIA) and albumin by immunoturbidimetric assay (COBAS 6000,8000 Roche, Switzerland) in serum. The parameters related to haemostasis were examined in plasma. The Clauss method determined the fibrinogen level, and the concentration of D-dimer, and vWF activity were measured using an immunoturbidimetric method (ACL TOP 350 CTS, ACL TOP 500 CTS Werfen, Spain). According to the manufacturer (Cloud-Clone Corp.), the ELISA test determined TF concertation.

### Statistical analysis

The normality of the distribution of the measured parameters was performed using the Shapiro–Wilk test. Medians (interquartile ranges) were used instead of means (SDs) if data were non-normally distributed.

The differences in the concentrations of 8-OHdG and AOPP between the COVID-19 moderate and severe groups were compared using the Mann–Whitney test and were considered significant at *P* < 0.05. The correlation between oxidative stress markers and other parameters was determined by Spearman’s test (r values) or Pearson’s test. The chi-square test and the chi-square with Yates’ correction were used to analyse the dependence of the occurrence of comorbidities in both study groups. All the analyses were performed with GraphPad Prism 9.0 (GraphPad Software, La Jolla, CA) and STATISTICA software (StatSoft, Inc., Kraków, Poland).

## Results

### Patients

The studied group of patients is highly homogenous, coming from the peak of the first wave of COVID-19 in Poland. The research group comprised 39 (54%) men and 33 (46%) women. The median age of the cases was 65 years (IQR 34–92 years). Among the patients with a past medical history, predominated hypertension (57%), obesity (34.7%), diabetes (23.6%), and atherosclerotic cardiovascular disease (ASCVD) (19.4%). In both analysed groups these conditions were the most common, and the other comorbidities differed slightly between the groups (chi-square test, *P* < 0.05; chi-square with Yates’ correction, *P* < 0.05).). The demographic data and comorbidities among the COVID-19 patients are summarised in (Table 1).

**Table 1.** Overall demographic and comorbidities presented at admission

Median parameter IQR Mean (SD)	Moderate group *n* = 25	Severe group *n* = 47	*P*-value
Sex *n* female/n male	13/12	20/27	0.4437
Age [years]	67 IQR (59–74)	67 IQR (55–72)	0.4603
Hypertension	16/25 64%	25/47 53%	0.3779
Diabetes type 2	5/25 20%	12/47 25.5%	0.5987
COPD	1/25 4%	2/47 4%	0.5702*
Asthma	2/25 8%	4/47 8.5%	0.7090*
CRF	2/25 8%	3/47 6%	0.8182*
HF	4/25 16%	9/47 44%	0.9929*
MF	2/25 8%	6/47 13%	0.8268*
Smokers	4/25 16%	6/47 13%	0.9841*
Obesity	11/25 44%	14/47 30%	0.2278
ASCVD	10/25 40%	17/47 36%	0.7493
Liver disease	1/25 4%	5/47 11%	0.6014*
Intubation	1/25 4%	9/47 19%	0.1580*
Without comorbidities	6/25 24%	8/47 17%	
Death	0	10/47 21%	

Abbreviations: ASCVD, atherosclerotic cardiovascular disease; CKD, chronic kidney disease; COPD, chronic obstructive pulmonary disease; CRF, chronic renal failure; HF, heart failure; MF, myocardial infarction.

* Chi-square with Yates’ correction.

### Patients with severe COVID-19 have higher levels of AOPP and 8-OHdG

**Figure 1.**
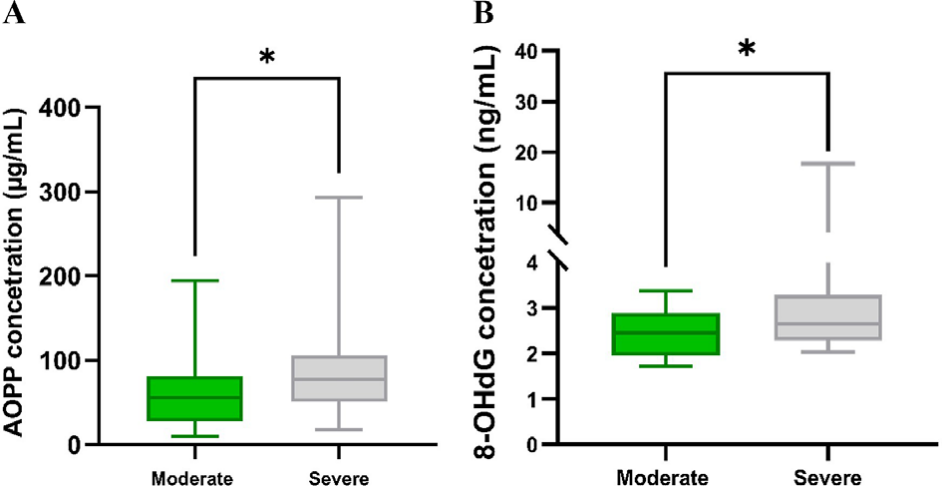
(a) Concentrations of AOPP in moderate (*n* = 25) and severe (*n* = 47) COVID-19 patients. (b) Concentrations of 8-OHdG in moderate (*n* = 25) and severe (*n* = 47) COVID-19 patients. Significant differences *P < 0.05.

We analysed two oxidative stress biomarkers, AOPP and 8-OHdG, in all 72 patients with COVID-19. Our results revealed differences in markers and their concentrations depending on the severity of the disease. The AOPP level in patients with severe COVID-19 [median 77.05 μg/mL (51.10–105.60)] was significantly higher (*P* = 0.0197) compared to moderate COVID-19 [median 56.21 μg/mL (27.85–81.66)] (Figure 1a). In parallel, there was a significant difference (*P* = 0.0435) in 8-OHdG concentration between severe [median 2.65 ng/mL (2.28–3.29)] and non-severe [median 2.45 ng/mL (1.94–2.89)] COVID-19 patients (Figure 1b).

### Patients with severe COVID-19 have lower levels of antioxidants and higher concentrations of inflammatory markers

Fibrinogen, zinc, vitamin D, iron, glucose, and albumin levels were measured. Fibrinogen concentrations were significantly raised, and albumin levels were significantly lowered in patients with severe COVID-19 when compared with moderate. However, there were no significant differences in iron, zinc, and vitamin D levels in both subgroups (Table 2).

**Table 2.** Laboratory tests

Parameters	Reference values	All patients (IQR)	Moderate patients (IQR)	Severe patients (IQR)	*P*-value Severe/moderate
Fe (μq/dL)	Women 37–145 Men 59–158	27 (21–41)	28 (24–41)	24 (19–42)	0.1892
Fibrinogen (mg/dL)	200–400	534 (412–657)	**434 (359–534)**	**577 (456–689)**	**0.0014**
Albumin (g/dL)	4.00–5.00	4 (3.6–4.2)	**4.2 (4.0–4.4)**	**3.9 (3.6–4.1)**	**0.0004**
Zinc (μq/dL)	70–114	86 (76–95)	91 (83–99)	84 (71–94)	0.0739
Vitamin D (nq/mL)	30–100	29 (18–45)	29 (24–40)	27 (17–48)	0.5868
Glucose	60–100	116 (106–137)	113 (104–136)	117 (106–145)	0.5084
TF (pg/mL)		433 (139–864)	**276 (92–641)**	**516 (179–972)**	**0.0398**
D-dimer (nq/FEU)	<500	932 (553–1644)	**420 (292–576)**	**1423 (878–2086)**	**<0.0001**
vWF activity (%)	depends on the patient’s blood group %	238 (201–394)	209 (191–347)	239 (207–411)	0.1087

Abbreviations: Fe, iron; TF, tissue factor; vWF, von Willebrand factor. Statistically relevant differences (p<0,05) are bolded.

### Patients with severe COVID-19 have higher levels of coagulation parameters

After measuring vWF, TF, and D-dimer, we detected statistically significant differences between the studied groups for the last two parameters. Higher concentrations of these markers of coagulopathy characterised patients with a severe course of COVID-19 (Table 2).

### Elevated Ratio AOPP/Alb in severe COVID-19 patients

**Figure 2.**
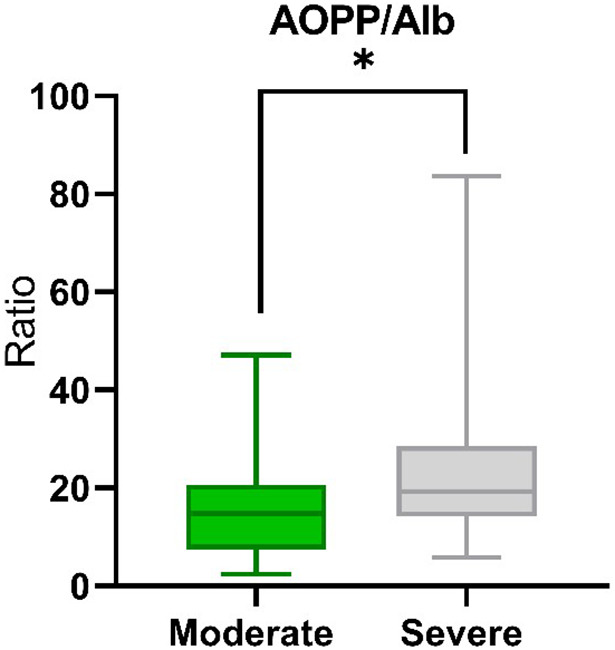
The median AOPP/Alb ratio in The median AOPP/Alb ratio in moderate (n=25) and severe (n=47) COVID-19 patients. Significant difference *P < 0.05.

We determined the ratio of AOPP to albumin, which turned out to be significantly different between the study groups. The median AOPP/Alb ratio for the moderate group was 14.81 and for the severe patient 19.33 (*P* = 0.0130) (Figure 2).

### Correlation

The next stage of the study was to examine the correlation between oxidative stress markers and other parameters measured in COVID-19 patients. Significant positive correlations were observed between measurements of AOPP and neutrophils (*r* = 0.2823), and the von Willebrand factor (vWF) (*r* = 0.3404) in all the patients. There were moderate correlations between levels of AOPP and vWF (*r* = 0.4238), and creatinine (*r* = 0.4163**)** in the group of moderate COVID-19 patients. Only in patients with moderate COVID-19 was a negative correlation between the measurements of AOPP and D-Dimer (*r* = −0.5108) observed. Significant correlations in the severe group of COVID-19 patients were detected between the concentrations of D-Dimer (*r* = 0.3049), glucose (*r* = 0.3111), and AOPP. The only significant negative correlation observed for 8-OHdG was with the iron in the moderate course group, but also in relation to the whole group of the patients (*r* = −0.3359).

## Discussion

COVID-19 is characterised by varying severity of the symptoms. However, despite intense research on the various aspects of the infection, there still need to be simple and quick markers predicting the course of this disease. Our study was the first to use the SCRI index for dividing patients into moderate/severe COVID-19 groups and the first to determine the level of oxidative stress markers based on that division.

The use of SCRI to divide patients by the course of infection revealed differences in the levels of oxidative stress parameters, suggesting that oxidative stress can also play a role in the pathogenesis of COVID-19. Previous studies have attributed the role of elevated levels of oxidative stress in the pathogenesis, progression, and severity of SARS-CoV-2 infection [12, 13]. Associated with virus replication and long-lasting SARS-CoV-2 infection, overstimulation of the immune response and ROS production lead to impaired system immunity responses [14]. Our results showed a statistically significant higher concentration of AOPP and 8-OHdG in the severe group compared to the moderate group of COVID-19 patients.

AOPPs are mainly described as aggregates of albumin, formed after exposure to free radicals, reflecting the degree of protein damage. An increase in both the number of neutrophils and the activity of these inflammatory cells leads to the release of myeloperoxidase (MPO) and the conversion of (H_2_O_2_) to hypochlorous acid (HOCL), which results in excessive formation of AOPP [15]. In our study, higher AOPP concentrations were also associated with higher neutrophil counts among all patients. Severe COVID-19 infection causes a lower respiratory capacity and low oxygen saturation of the blood, which leads to a state of hypoxia [16]. Furthermore, acute hypoxia is accompanied by an increase in the formation of ROS and AOPP [17]. An increased level of AOPP is often observed with the worsening of the patient’s ongoing pathological conditions [14, 18]. Ducastel at el. [19] documented the induction of AOPP with increasing severity of the course of COVID-19. Interestingly Kosanovic et al. [20] showed that hospitalised COVID-19 patients had higher AOPP concentrations than non-hospitalised patients. Their results indicate that the longer the patient stays in the hospital, the higher the AOPP levels.

Elevated AOPP levels have been observed in early renal failure, consistent with increased creatinine levels [8]. It has also been suggested that increased levels of AOPP may contribute to kidney damage [21]. Our research has shown that in patients with moderate COVID-19, the higher the AOPP level, the higher the creatinine level.

The effect of AOPP on coagulopathy in COVID-19 should also be considered. We demonstrated that higher concentrations of clotting components such as vWF, TF, and D-dimer accompany increasing concentrations of AOPP.

Oxidative stress changes the membrane potential of platelets and impairs ATP production, which in the aftermath conjures up excessive platelet activation. AOPP promotes platelet activation by CD36 and thus may induce intracellular signalling, including a vicious cycle of ROS production [22].

During the COVID-19 pandemic, the vWF became a specific marker of endothelial dysfunction, helpful in determining the severity of the disease [23]. vWF is involved in primary and secondary haemostasis. We observed that all participants and patients with moderate COVID-19 showed higher vWF activity as AOPP levels increased.

Another component that was associated with higher concentrations of advanced oxidative protein products in severely ill patients was D-Dimer. The meta-analyses showed that increased levels of D-Dimer were associated with a worse prognosis for COVID-19 [24]. The negative correlation observed in patients with moderate COVID-19 can be explained by the fact that coagulopathy and oxidative stress processes are not as developed and stimulated as in patients with a severe course of this disease. We noted that increased TF levels in severe patients were associated with higher AOPP.

It was found that oxidised albumin may be a direct mediator of triggering inflammation and activating neutrophils, thus possibly exacerbating oxidative stress [6]. NET (extracellular neutrophil traps) is involved in thrombogenic activity. It was shown that the neutrophils of the tested COVID-19 patients displayed a high expression of TF, and in addition, their networks secreted active TF [25]. More coagulation parameters suggestive of disturbances were correlated with oxidative stress markers in the severe group than in the moderate group, confirming our assumptions.

The second tested biomarker, 8-hydroxy-2′-deoxyguanosine, reflects damage to DNA. Our research showed that as the severity of the disease progresses, so does the level of 8-OHdG. During infection, the cumulative damage at the DNA level caused by the presence of SARS-CoV-2 results in the modification of macromolecules, which affects the progression of the disease. Moreno-Fernandez et al. [14] studied pregnant women with COVID-19, and this population displayed a higher concentration of 8-OHdG when compared to healthy individuals. A similar observation was made by Tantry et al. [7], who noted higher 8-OHdG in patients with events such as stroke, myocardial infarction, and pulmonary embolism versus those without these diagnoses.

In our study, the only relationship between the level of 8-OHdG and other analysed parameters concerned the concentration of iron. Oxidant properties are attributed to iron due to its ability to reduce oxygen to superoxide radicals at both intracellular and extracellular levels. In addition, iron collected in ferritin can impact the direct initiation of lipid peroxidation [26]. Dysregulated iron metabolism is characteristic of COVID-19 patients. According to data, high ferritin levels and anaemia may contribute to multiple organ failures in the disease [27]. Inflammation also causes increased secretion of hepcidine, which contributes to hypoferremia [28]. Our study showed that iron levels in the test sera were almost two times below reference values regardless of the patient’s clinical condition. Ferroptosis caused by excess iron accumulated in cells results from the oxidative stress cascade. This is the connecting point between low iron circulating concentrations among patients and increased levels of 8-OHdG.

We found significant differences in fibrinogen levels between the tested groups. Higher fibrinogen concentrations characterised patients with a severe infection course, but this is to be expected as the infection is accompanied by cytokine storm and fibrinolytic activation. An increase in fibrinogen may not only indicate an exacerbation of inflammation but also aggravate coagulopathy [29].

Our study also included factors with antioxidant properties: vitamin D, zinc, and albumin. Only the albumin concentration showed a significant difference between the study groups. It is well known that albumin is a negative acute-phase protein (APP) for blood osmotic pressure and ligands transportation. Albumin also supports antioxidant defenses in circulation. Hypoalbuminemia is linked to hypercoagulability, which can lead to thrombosis and even death in COVID-19 [30]. It has even been reported that the infusion of albumin with antiplatelet drugs may be one of the best solutions for anticoagulant therapy in critically ill patients with SARS-Cov-2 infection [31]. Our results showed that non-severe patients had a significantly increased albumin level compared to severe COVID-19 patients. This suggests that in cases of moderate infection, the body’s ability to defend itself against oxidative stress is higher when compared to more severe cases.

Decreased vitamin D and zinc levels are associated with more severe COVID-19, extended hospitalisation, and severe acute pneumonia [32, 33]. We found no significant relationship between the concentration of vitamin D and zinc and the course of the disease. Still, almost all patients were characterised by a significantly reduced concentration of these vitamins.

The last element of our study was the comparison of the AOPP/Alb ratio (proportion of advanced protein oxidation products/albumin) between the study groups. We found significant differences in the value of this indicator (*P* = 0.0130) depending on the course of the patient’s disease. Wybranowski et al. [6] also noticed that this coefficient is positively correlated with D-Dimer concentrations in COVID-19 patients, which indirectly indicates that the higher the index, the more severe the disease.

In conclusion, severe Covid-19 patients with SCRI index <1.19 have higher concentrations of AOPP and 8-OHdG than the moderate group. The AOPP/Alb ratio is higher and also characteristic of patients with severe COVID-19, and perfectly reflects the balance between oxidative stress and the antioxidant capacity of the human body. It leads us to conclude that oxidative stress during COVID-19 may be responsible for the worse course of the disease. Moreover, coagulation disorders, inflammation, and increasing oxidative stress in COVID-19 patients form three interconnected links that affect each other.

## Data Availability

The data that support the findings of this study are available upon request from the corresponding author.
